# Semi-Centennial Meeting of the Mississippi Valley Dental Association

**Published:** 1895-09

**Authors:** 


### Proceedings.

### Semi-Centennial Meeting of the Mississippi Valley Dental Association.

FIRST DAY—MORNING SESSION.

The fiftieth annual session of the Mississippi Valley Association of Dental Surgeons convened in Hall E, of the Odd Fellows' Temple, corner of Seventh and Elm Streets, Cincinnati, O., on Wednesday morning, April 17th, at 8 o'clock, with the President Dr. J. Taft, of Cincinnati, in the Chair.

The proceedings of the convention were opened with prayer by Dr. Taft. Minutes of the forty-ninth session were read and approved.

The Treasurer submitted his report, showing a balance in the Treasury of $49.59. Same referred to an Auditing Committee, composed of Drs. C. M. Wright and H. C. Matlack, who, after examining and auditing the same, reported all correct.

On motion of Dr. Callahan, the Executive Committee was authorized to secure the services of Douglas A. Brown, stenographer, to report the proceedings of the convention.

The President named as a committee to draft resolutions of condolence upon the death of Dr. J. J. R. Patrick, of Belleville, Ills., Messrs. Drs. Harlan and Betty.

Dr. George Evans, of New York City, briefly addressed the Convention, and was followed by Drs. Harry Benedict Respinger and Edmund Dubuis, of Geneva, Switzerland. These gentlemen were present by invitation, and remained throughout interested participants in the meetings. These remarks were of a general and congratulatory character, and are not reported at length because of their having been delivered prior to the arrival of the stenographer. President Taft then relinquished the Chair temporarily to First Vice-President Ames, of Chicago.

The President's address being next in order was then delivered, giving some suggestion on various points of interest which will be found on page 214 Register of May.

